# Anatomical study of type classification and surface area of attachment sites for tibialis anterior tendon

**DOI:** 10.1186/s12891-023-06753-8

**Published:** 2023-08-03

**Authors:** Tomoki Hirai, Mutsuaki Edama, Ryoya Togashi, Haruki Osanami, Rina Saito, Koyo Kato, Mayuu Shagawa, Chie Sekine, Hirotake Yokota, Ryo Hirabayashi, Tomonobu Ishigaki, Hiroshi Akuzawa, Yuki Yamada, Taku Toriumi, Ikuo Kageyama

**Affiliations:** 1 Institute for Human Movement and Medical Sciences, Niigata University of Health and Welfare, Shimami-Cho, 1398, Kita-Ku, Niigata City, Niigata 950-3198 Japan; 2 Department of Anatomy, School of Life Dentistry at Niigata, Nippon Dental University, Niigata, Japan

**Keywords:** Tibialis anterior tendon, Attachment site area, Three-dimensional, Medial cuneiform bone, First metatarsal bone

## Abstract

**Background:**

The purpose of this study was to clarify the attachment types of the tibialis anterior tendon (TAT) in Japanese fixed cadavers and to determine the attachment site area in three dimensions.

**Methods:**

We examined 100 feet from 50 Japanese cadavers. The TAT was classified according to differences in the number of fiber bundles as: Type I, with one fiber bundle; Type II, with two fiber bundles; and Type III, with three fiber bundles. The attachment site area of the TAT was measured using a three-dimensional scanner.

**Results:**

Cases were Type II in 95% and Type III in 5%, with no cases of Type I identified. In Type II, mean attachment site areas were 85.2 ± 18.2 mm^2^ for the medial cuneiform bone (MCB) and 72.4 ± 19.0 mm^2^ for the first metatarsal bone (1 MB), showing a significantly larger area for MCB than for 1 MB.

**Conclusions:**

These findings suggest the possibility of ethnic differences in TAT attachment types and suggest that TAT attachments in Japanese individuals are highly likely to be Type II, with rare cases of Type III. Accurate measurement of attachment site areas is possible with appropriate three-dimensional measurements.

## Background

The tibialis anterior muscle (TA) is a characteristic structure divided into two parts: anterior fiber bundles and posterior fiber bundles. The anterior fiber bundles of the TA originate from the anterior border of the tibia and the lateral condyle. In contrast, the posterior fiber bundles of the TA originate from the anterolateral surface of the tibia and the anterior surface of the interosseous membrane. The TA transitions to a flat tendon while descending to the anterolateral surface of the tibia, passing under the extensor retinaculum at the anterior, lower end of the lower leg, then attaching to the medial cuneiform bone (MCB) and first metatarsal bone (1 MB) [1]. The function of the TA is inversion and dorsiflexion of the ankle joint and it is involved in maintaining the medial longitudinal arch [2]. During motion, the TA is active at heel strike and during the swing phase to control foot drop and prevent tripping [3]. The activity of the TA increases with walking speed and decreases with the switch to running [3]. The TA is one of the most important muscles in daily life because it is deeply involved in human movement [4, 5].

Rupture of the TA tendon (TAT) is a rare injury that is commonly diagnosed late due to mild clinical signs and symptoms (such as mild pain, localized mass, palpable gap, loss of strength, instability during gait, gait disturbances with forefoot dropping and stumbling, and high stepping gait) [6]. Previous studies [7–9] reported that despite the disruption of the TAT being a rare condition, this pathology is the third most common form of tendon rupture in the lower limb, after the Achilles tendon and patellar tendon. Surgical reconstruction of the TAT (such as tendon transfer, repair of all muscle, or allograft augmentation) is the treatment of choice in cases with severe impairment of dorsal extension and supination of the foot [10–13]. Different techniques have been reported according to the severity of tendon injury or gap formation. To restore the natural lever arm of the TA, the TAT must be reinserted at the anatomical attachment site [8]. Therefore, precise anatomical description of ligament and tendon attachments is crucial and can help optimize reconstruction procedures in terms of anchor placement and graft sizing [14].

The anatomy of the TAT has long been reported in previous studies using cadavers. Regarding the attachment of the TAT to bone, types with one, two, and three fiber bundles have been reported [10, 11, 15–19], with the two-bundle type reported as the most frequent [10, 15, 16, 18, 19]. Regarding the type with a single bundle of fibers, Musial et al. [18] and Willegger et al. [10] found no types with a single fiber bundle in their studies of 122 and 41 fixed cadavers, respectively. However, Karauda et al. [17] examined 100 pairs of fetal fixed cadavers and reported the one fiber bundle type as the most frequent, at 60%. For the type with three fiber bundles, Brenner [15] and Willegger et al. [10] reported that these were not present, while Olewnik et al. [16] and Karauda et al. [17] reported frequencies of 2% and 4%, respectively. Such findings suggest that the frequencies of TAT attachment types remain controversial. In addition, it suggested that TAT attachment types may vary by ethnicity (Caucasian [16], European [17], Not Mentioned [10, 11, 15, 18, 19],). Further, very few studies have examined the footprint (attachment site area) of the TAT, and most studies are limited to examinations of the width and thickness of the TAT and the shape of the footprint [10, 11, 15–19]. A previous study [10] examining the footprint of the TAT reported that the mean attachment site area to MCB was 71.5 mm^2^ (range, 20.1–151.0 mm^2^) and that to 1 MB was 48.1 mm^2^ (range, 18.5–97.0 mm^2^) for the type with two bundles of the TAT. However, measurement of the attachment site area in that previous study [10] was limited to two-dimensional measurement. Since the surfaces of the MCB and 1 MB, as the attachment sites of the TAT, comprise curved surfaces, measurement of attachment surface areas should be performed in three dimensions.

The purpose of this study was to clarify the attachment types of the TAT in Japanese fixed cadavers and to determine the attachment site area in three dimensions. The hypothesis for this study was that the proportions of type classifications would differ from those of previous studies, and that the area of attachment to bone would be larger than in previous studies.

## Methods

### Cadavers

This investigation examined 100 feet from 50 Japanese cadavers (mean age at death, 80 ± 11 years; 56 sides from 28 men, 44 sides from 22 women; 50 right sides, 50 left sides) that had been switched to alcohol after placement in 10% formalin. No feet showed any sign of previous major surgery around the foot or ankle. This study was conducted with the approval of the ethics committee at our institute (approval number: 18867). Informed consent for the storage and use of the bodies for research purposes was given by the donors prior to their deaths or by their next of kin.

### Methods

**Fig. 1:**
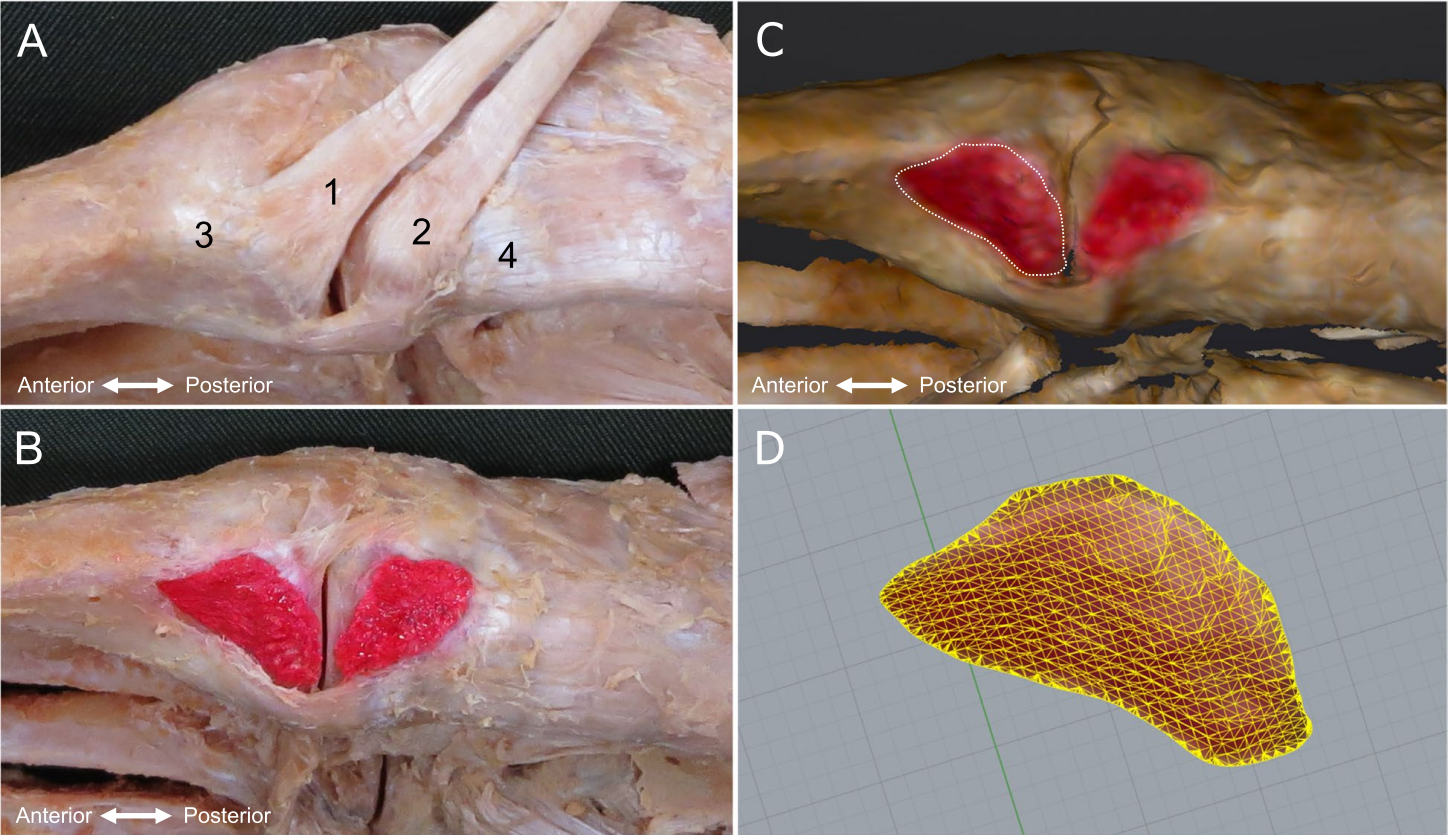
Method for measuring attachment site area. **A** Attachment site of tibialis anterior tendon: right foot, medial view. **B** Attachment site was identified by peeling off adherent tissue, then coloring the site with red pencil. **C** Using a 3D scanner to make a 3D foot sample. A curve was drawn as the boundary of the attachment site for the first metatarsal bone with a pen-type device. **D** Surface area of attachment was calculated using Rhinoceros 3D software. 1: TAT fiber bundle insertion into the base of the first metatarsal; 2: TAT fiber bundle insertion into the medial cuneiform; 3: first metatarsal bone; 4: medial cuneiform bone

The dissection procedure for the TAT is described below. First, isolated specimens of the leg were created by transection about 10 cm above the ankle joint. Skin, subcutaneous tissue, and muscle were removed, the TAT was carefully dissected out, and the attachment to the bone was confirmed (Fig. 1a). The TAT was classified according to differences in the number of fiber bundles as macroscopic: Type I, with one fiber bundle; Type II, with two fiber bundles; and Type III, with three. The attachment site area was measured with reference to the previous study[20]. The attachment site area was identified by peeling away the TAT attachments and the periosteum, then coloring the attachment site with a red pencil (Fig. 1b). The surface area was then measured using a three-dimensional (3D) scanner (EinScan Pro HD, measurement precision according to manufacturer, 0.04 mm; SHINING 3D, Hangzhou, China) to produce a 3D foot sample. The resulting data were read into Geomagic Freeform 2021 design software (3D SYSTEMS), and the boundary of the attachment site was drawn as a curve with a pen-type device (Touch; 3D SYSTEMS) (Fig. 1c). Surface area was then calculated using Rhinoceros7 3D software (McNeel) (Fig. 1d). All measurements were performed by the same physical therapist (T.H.).

The reliability of surface area measurement by 3D scanner was calculated using the intraclass correlation coefficient (ICC) (1,1) for 10 of the 100 feet. The retest of the surface area measurement was performed the same day because ink bled over time and could be overestimated during digitization. The ICC (1,1) for the measurement was 0.98. According to the criteria of Landis and Koch. [21], reliability is considered “almost perfect” for ICCs of 0.81 or more, so the reliabilities of attachment site area measurements in this study were considered almost perfect.

### Statistical analysis

Statistical analyses were performed using SPSS version 24.0 (SPSS Japan, Tokyo, Japan). For Type II (with two fiber bundles), the difference in attachment site area between the fiber bundle attached to the MCB and that attached to the 1 MB was compared using paired t-tests. A significance level of 5% was used.

## Results

### Type classification by number of TAT fiber bundles

**Fig. 2:**
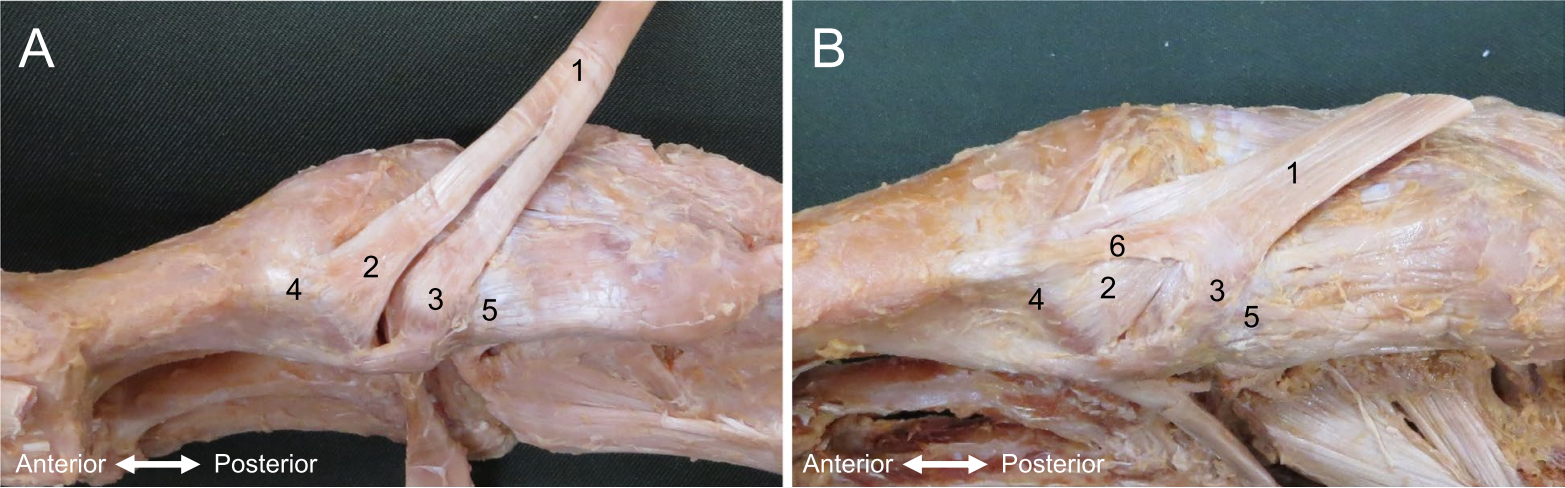
Type classification by number of TAT fiber bundles. **A** Type II TAT with two fiber bundles: right foot, medial view. **B** Type III TAT with three fiber bundles: right foot, medial view. 1: Tibialis anterior tendon; 2: TAT fiber bundle insertion into the base of the first metatarsal; 3: TAT fiber bundle insertion into the medial cuneiform; 4: first metatarsal bone; 5: medial cuneiform bone; 6: TAT fiber bundle insertion into the base of the first metatarsal

No feet showed Type I (with one fiber bundle). Type II (with two fiber bundles) was seen in 95 legs (95%) and Type III (with three fiber bundles) was seen in 5 legs (5%) (Fig. 2). Type II was attached to MCB and 1 MB in all cases (95 legs). Type III was attached to the MCB (one fiber bundle) and 1 MB (two fiber bundles) in all cases (5 legs).

### Surface area of TAT attachment site

In Type II, mean attachment site area for the MCB was 85.2 ± 18.2 mm^2^, and that for 1 MB was 72.4 ± 19.0 mm^2^. Mean attachment site area to MCB was significantly larger than that to 1 MB (p < 0.01). Statistical analysis was not performed for Type I and Type III due to the small number of samples.

## Discussion

The purpose of this study was to clarify the attachment types of the TAT in Japanese fixed cadavers and to determine 3D attachment site areas. To the best of our knowledge, this represents the first study to clarify the type of classification by number of TAT fiber bundles using Japanese cadavers and 3D attachment site area.

In this study, for type classification by the number of TAT fiber bundles, Type I (with one fiber bundle) was not present, while Type II (with two fiber bundles) was present in 95 legs (95%) and Type III (with three fiber bundles) in 5 legs (5%). Type II was definitively the most common result. Type classification by the number of TAT fiber bundles has long been reported in many cadaveric studies [10, 11, 15–19]. For Type I, Olewnik et al. [16] examined the attachment site area of the TAT using 100 fixed Caucasian cadavers and reported that 32 cadavers (32%) showed a single bundle of fibers. Karauda et al. [17] also examined TAT attachment sites using 100 European fetal remains and reported that 60 fetuses (60%) showed a single bundle of fibers, a result differing markedly from that of the present study. As for Type II, other than the previous study using European fixed fetal cadavers [17], the type with two bundles was uniformly reported as the most common, as in the present study [10, 15, 16, 18, 19]. Type III was reported as either absent [10, 15] or present in only very small numbers (2–4%) [16, 17]. Furthermore, variations in the attachment of foot and ankle muscles such as the tibialis posterior [20], peroneus longus [22], and flexor hallucis longus [23] may vary by ethnicity. The present findings thus suggest the possibility of ethnic differences in TAT attachment types and suggest that the TAT attachment type in Japanese is highly likely to be Type II (with two fiber bundles), with rare cases of Type III (with three fiber bundles). Differences from fetal specimens will require further research in the future.

Mean attachment site areas for the TAT in the present study were 85.2 ± 18.2 mm^2^ and 72.4 ± 72.4 mm^2^ for MCB and 1 MB, respectively, showing a significantly larger area of fiber bundle attachment to the MCB. Willegger et al. [10] measured the attachment site area of the TAT by photographing the specimen with a camera then making measurements in two dimensions using Image J image analysis software. They reported the attachment site areas for the TAT as 71.5 mm^2^ (range, 20.1–151.0 mm^2^) and 48.1 mm^2^ (range, 18.5–97.0 mm^2^) for MCB and 1 MB, respectively, again showing a larger attachment site area for MCB than for 1 MB. Iwama et al. [24] examined the attachment site area of the anterior cruciate ligament in the knee using 39 cadaveric knees. They stated that a 3D camera or computer modeling software could evaluate the attachment site area more appropriately. In addition, thin-slice 3D MRI [25] and 3D computed tomography imaging [26] have been reported to help reconstruct the 3D ATFL model to provide accurate anatomical knowledge of the area and location of ATFL. The attachment site area of the TAT lies along curved and uneven surfaces of bone, so two-dimensional measurements are unlikely to accurately capture the surface area. The attachment site area in the present study was therefore larger than in previous studies, and more accurate measurement of attachment site area was possible because of the appropriate 3D measurements. After surgical repair, anatomic reconstruction of the natural course and biomechanical lever arm should be pursued to restore dorsal extension power and forefoot supination [10]. The findings of this study of knowledge of the size and location of the footprint are helpful in surgical decision-making.

Some limitations need to be considered when interpreting the findings from this study. First, since the same subject's left and right feet are included in the dataset as independent subjects, the significance level of the difference may be overestimated. Second, as all cadavers were from Japanese individuals, whether the present findings apply to individual from other ethnicities is unclear. Further studies are required to evaluate variations according to ethnic origin. Third, in this study, only the attachment site and surface area of the TAT were examined, and detailed attachment sites and attachment shapes were not examined. In the future, the effects of different attachment types and attachment site areas and shapes of the TAT in vivo on TAT function will need to be examined.

## Conclusions

The findings of the present study suggest the possibility of ethnic differences in TAT attachment types and suggest that the TAT attachment type in Japanese is most likely to be Type II (with two fiber bundles), with rare cases of Type III (with three fiber bundles). Attachment site area was larger than in previous studies, with more accurate measurement of attachment site area possible with appropriate 3D measurements.

## Data Availability

The data that support the findings of this study are available from the corresponding author upon reasonable request.
